# Sepsis caused by *Phytobacter diazotrophicus* complicated with galactosemia type 1 in China: a case report

**DOI:** 10.1186/s12879-024-09458-y

**Published:** 2024-06-19

**Authors:** Jiansheng Lin, Junfeng Wu, Lan Gong, Xiaoqing Li, Gaoxiong Wang

**Affiliations:** 1 Microbiology Laboratory, Quanzhou Women’s and Children’s Hospital, Quanzhou, People’s Republic of China; 2 Infection Disease Department, Quanzhou Women’s and Children’s Hospital, Quanzhou, People’s Republic of China; 3 St George and Sutherland Clinical Campus, UNSW Microbiome Research Centre, University of New South Wales, Sydney, NSW 2052 Australia; 4 Neonatal Department, Quanzhou Women’s and Children’s Hospital, Quanzhou, People’s Republic of China; 5 Research Administration Office, Quanzhou Women’s and Children’s Hospital, Quanzhou, People’s Republic of China; 6 The Affiliated Women’s and Children’s Hospital of Huaqiao University, Quanzhou, People’s Republic of China; 7 School of Medicine, Huaqiao University, Quanzhou, 362021 China

**Keywords:** *Phytobacter diazotrophicus*, Sepsis, Galactosemia type 1, Neonatal

## Abstract

**Background:**

*Phytobacter diazotrophicus* (*P*. *diazotrophicus*) is an opportunistic pathogen that causes nosocomial outbreaks and sepsis. However, there are no reports of *P. diazotrophicus* isolated from human blood in China.

**Case presentation:**

A 27-day-old female infant was admitted to our hospital with fever and high bilirubin levels. The clinical features included jaundice, abnormal coagulation, cholestasis, fever, convulsions, weak muscle tension, sucking weakness, ascites, abnormal tyrosine metabolism, cerebral oedema, abnormal liver function, clavicle fracture, and haemolytic anaemia. The strain isolated from the patient’s blood was identified as *P. diazotrophicus* by whole-genome sequencing (WGS). Galactosemia type 1 (GALAC1) was diagnosed using whole-exome sequencing (WES). Based on drug sensitivity results, 10 days of anti-infective treatment with meropenem combined with lactose-free milk powder improved symptoms.

**Conclusion:**

*P. diazotrophicus* was successfully identified in a patient with neonatal sepsis combined with galactosemia. Galactosemia may be an important factor in neonatal sepsis. This case further expands our understanding of the clinical characteristics of GALAC1.

## Introduction

*Phytobacter diazotrophicus* (*P*. *diazotrophicus*), a gram-negative bacterium, is an opportunistic pathogen [1]. In 2008, *P. diazotrophicus* was first described as an endophytic enterobacterial species isolated from wild rice in China [2]. In Brazil, *P. diazotrophicus*-contaminated total parenteral nutrition has led to infection outbreaks in neonatal intensive care units [3]. Although *P*. *diazotrophicus* has only been recently described in human samples [4, 5], WGS revealed that it has been frequently misidentified as *Pantoea*, *Metakosakonia* or *Kluyvera* since the 1970s [3, 5]. *Pantoea* spp. (later corrected to *Phytobacter* spp [5]. ) outbreaks have resulted in high neonatal mortality [6]. To date, *P. diazotrophicus* has not been isolated from blood in China.

Galactosemia is an autosomal recessive inherited metabolic disorder of galactose metabolism. GALAC1 is caused by mutations in the *GALT* gene mapped to chromosome 9p13; it is the most frequent form of galactosemia, with a prevalence of approximately 1 in 60,000 live births in the general population [7].

Here, we report a rare case of neonatal sepsis caused by *P. diazotrophicus* combined with GALAC1.

## Case presentation

A 27-day-old female infant who presented with fever half a day ago and high bilirubin levels was admitted to hospital. She was delivered vaginally at 37^+ 3^ weeks, with no history of resuscitation for asphyxia, birth weight of 2680 g, and Apgar score of 10-10-10. After birth, she was admitted to a local county hospital for neonatal hyperbilirubinemia, disseminated intravascular coagulation, and neonatal ABO haemolytic jaundice. During the 20 days of initial hospitalization, she received treatments such as infusion of human immunoglobulin, human albumin, fresh frozen plasma, low molecular weight heparin sodium, cryoprecipitation coagulation factors, and leukoreduced red blood cells. At 4 days old, tandem mass spectrometry was performed for neonatal genetic metabolic disease screening and revealed increased tyrosine, phenylalanine, and citrulline levels, which were ignored during the initial hospitalization.

Upon physical examination, her body temperature, blood pressure, pulse rate, and respiratory rate were 37.5°C, 88/44 (55) mmHg, 150 beats/min, and 52 breaths/min, respectively. The infant exhibited yellowish skin, muscle weakness, limb convulsions, an incomplete hugging reflex, and a weak sucking reflex. The laboratory results were as follows: low blood glucose (1.3 mmol/L), high neonatal total bilirubin (340 umol/L), high direct bilirubin (229.9 umol/L), high total bile acid (201.3 umol/L), marginally elevated alanine aminotransferase (53 U/L), marginally elevated aspartate aminotransferase (73 U/L), elevated C-reactive protein (18.43 mg/L; normal range:≤1.6 mg/L), elevated interleukin-6 (109 pg/mL; normal range:18–26 pg/mL), elevated procalcitonin test (11.74 ng/mL; normal range: ≤0.5 ng/mL), prolonged prothrombin time (> 120 sec), decreased fibrinogen (< 0.5 g/L), prolonged activated partial thromboplastin time (> 180 sec), and decreased haemoglobin (87 g/L). The direct antiglobulin test (Coombs’) results were positive for anti-human IGG and negative for anti-human C3. Urinalysis revealed +++ urinary bilirubin. Cerebrospinal fluid analysis was normal. Anteroposterior chest radiograph revealed a left clavicle fracture. Routine colour ultrasound of the infant’s belly revealed a slightly enlarged liver, thickened gleasonian sheath, enhanced parenchymal echo of both kidneys, and large amounts of ascites. Cranial colour ultrasonography revealed cerebral oedema. Using a video electroencephalogram, focal epileptic electrical seizures were detected, with numerous left occipital area waves observed between ictal statuses.

**Fig. 1:**
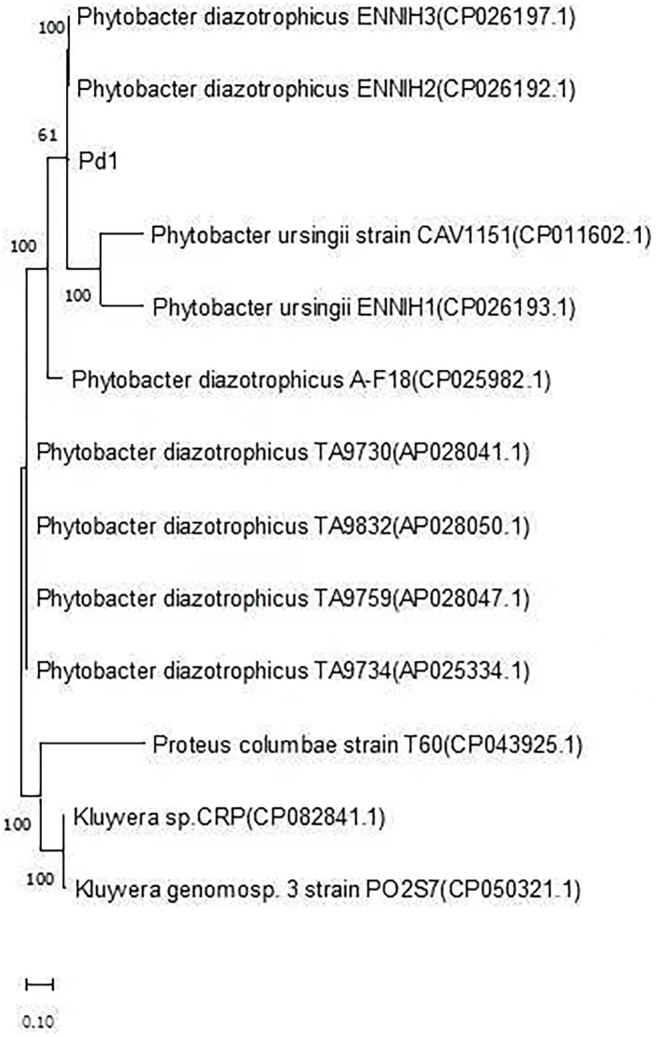
Phylogenetic analysis between the strains. The *strains* genomes available on the NCBI database and Pd1 isolated in this study

On the third day of hospital admission, two blood cultures from two different body parts revealed gram-negative rods; therefore, neonatal sepsis was considered. Anti-infective treatment with piperacillin/tazobactam was administered before culture report. VITEK-2 GN card (bioMérieux) displayed acceptable identification of *Kluyvera* intermedius (93%). The MALDI Biotyper-Bruker Microfex showed that *Phytobacter ursingii*, which has a spectral score of 1.9 (low confidence), was the closest match, and the strain was subjected to WGS. Multilocus sequence analysis (MLSA) concatenated with five housekeeping genes (dnaJ, mdh, pyrC, recA, and rpoD), which is an effective method for identifying bacterial species, was used to perform phylogenetic analyses, using the MEGA11 software. The closest affinity of Pd1 was with *P. diazotrophicus* ENNIH2 and ENNIH3 (Fig. 1). To further identify the strain, genomic relatedness among different species was determined using web-based DNA-DNA hybridisation, including in silico DNA-DNA hybridisation (dDDH) and average nucleotide identity (ANI). The dDDH and ANI values for strains Pd1, *P. diazotrophicus* ENNIH2, *P. diazotrophicus* ENNIH3, *P. diazotrophicus* A-F18, *P. diazotrophicus* TA9759, and *P. diazotrophicus* TA9832 ranged from 78.5 to 99.9% and 97.5–99.9%, respectively (Table 1 and Table 2; above the 70 and 95% thresholds), which indicated that strain Pd1 was *P. diazotrophicus*. Finally, in this study, we determined that the causative neonatal sepsis pathogen was *P. diazotrophicus* and not *K. intermedius*, as reported by the laboratory.

**Table 1:** Digital DDH of strains using genome-to-genome distance calculator

Strains	Pd1	ENNIH2	ENNIH3	A-F18	TA9759	TA9832	ENNIH1	CAV1151	CRP	PO2S7
Pd1	100									
*P. diazotrophicus* ENNIH2	78.9	100								
*P. diazotrophicus* ENNIH3	78.9	99.9	100							
*P. diazotrophicus* A-F18	79.0	89.9	89.9	100						
P. *diazotrophicus* TA9759	79.9	90.6	90.6	90.8	100					
*P. diazotrophicus* TA9832	78.5	89.4	89.4	88.7	99.0	100				
*P. ursingii* ENNIH1	46.5	46.8	46.8	47.2	46.9	46.8	100			
*P. ursingii* strain CAV1151	45.4	45.5	45.5	46.2	45.5	45.1	70	100		
*Kluyvera* sp. CRP	22.2	22.4	22.4	22.5	22.4	22.3	22.4	22.4	100	
*Kluyvera* genomosp. 3 strain PO2S7	22.1	22.2	22.2	22.2	22.2	22.0	22.1	22.2	90.4	100

*Note* DDH values of over 70% are shaded

**Table 2:** Mean ANI values for all available genomes of strains

Strains	Pd1	ENNIH2	ENNIH3	A-F18	TA9759	TA9832	ENNIH1	CAV1151	CRP	PO2S7
Pd1	100									
*P. diazotrophicus* ENNIH2	97.5	100								
*P. diazotrophicus* ENNIH3	97.6	99.9	100							
*P. diazotrophicus* A-F18	97.6	98.8	98.8	100						
*P. diazotrophicus* TA9759	97.7	98.9	98.9	98.9	100					
*P. diazotrophicus* TA9832	97.5	98.8	98.7	98.8	99.9	100				
*P. ursingii* ENNIH1	92.1	92	92	92.2	92.2	92.1	100			
*P. ursingii* strain CAV1151	91.8	91.8	91.8	91.9	91.8	91.8	96.3	100		
*Kluyvera* sp. CRP	78.3	78.3	78.4	78.6	78.4	78.0	78.5	78.2	100	
*Kluyvera* genomosp. 3 strain PO2S7	78.2	78.4	78.4	78.3	78.3	77.9	78.1	78.2	98.8	100

*Note* ANI values over 95% are shaded

Based on the combination of the tandem mass spectrometry results and clinical features, inherited metabolic diseases need to be considered. Therefore, WES was performed for the patient and her parents to identify the mutations in the patient. Two pathogenic/likely pathogenic compound heterozygous mutations were identified: a nonsense mutation NM_000155: c.610 C > T:p. Arg204Ter in exon7 (pathogenic) and a missense mutation NM_000155: c.1034 C > A:p. Ala345Asp in exon10 (likely pathogenic) of *GALT* gene, which explains the patient’s clinical phenotype. The patient’s parents were heterozygous carriers of both mutations. Compound heterozygous mutations in *GALT* were consistent with the autosomal recessive inheritance pattern of GALAC1. Therefore, based on confirmed GALAC1, the newborn was switched to lactose-free milk powder.

The antimicrobial susceptibility of the isolated strain was determined using the VITEK 2 bacterial antibiotic sensitivity analysis system (bioMérieux). The results were interpreted based on breakpoints recommended by the Clinical and Laboratory Standards Institute M100-S26 for Enterobacteriaceae. Based on the antimicrobial susceptibility results (Table 3), the antimicrobial therapy was changed to meropenem. Meropenem was administered for 10 days to fight the infection until the infection index returned to the normal range. The 22-day comprehensive treatments also included the multiple transfusion of red blood cells, filtered fresh frozen plasma, cryoprecipitate coagulation factors, fibrinogen, albumin gamma globulin, and low molecular weight heparin, dehydration and diuresis to reduce intracranial pressure, ursodeoxycholic acid to protect the liver and gallbladder, ascites drainage, intravenous nutrition, and phenobarbital sedation maintenance.

**Table 3:** Antibiotic resistance result for strain Pd1

Antibiotics	MIC (µg/mL)	Interpretation
Amoxicillin/clavulanic acid	≤2	S
Piperacillin/tazobactam	≥ 128	R
Cefuroxime	4	S
Cefuroxime Axetil	4	S
Cefoxitin	8	S
Ceftazidime	1	S
Ceftriaxone	≥ 64	R
Cefoperazone/sulbactam	≤ 8	S
Cefepime	≤ 0.12	S
Ertapenem	≤ 0.12	S
Imipenem	≤ 0.25	S
Amikacin	≤ 2	S
Levofloxacin	≤ 0.25	S
Tigecycline	≤ 0.5	S
Cotrimoxazole	≤ 20	S

*Abbreviation* S, susceptible; R, resistant; MIC, Minimum Inhibitory Concentration

## Discussion and conclusions

In this study, we found that phenotypic identification methods could not identify *P. diazotrophicus*, which is consistent with a previous report [4]. The in-house SuperSpectrum for MALDI-TOF MS can identify *Phytobacter* spp [5], however, we repeatedly failed to successfully identify *P. diazotrophicus*, possibly because we used instruments from different manufacturers. WGS is the most reliable approach for identifying suspected isolates at the species level using ANI, dDDH, or core genome phylogeny [5].Through ANI, dDDH, and MLSA phylogeny, *P. diazotrophicus* was successfully identified.

The OMIM database (No.230400) indicated a high incidence of *E. coli* sepsis in untreated neonates GALAC1 [8], suggesting that galactosemia may be an important factor involved in sepsis. It is worth noting that the isolate in this study was not *E. coli* but *P. diazotrophicus*, which facilitated the identification of the GALAC1 clinical features. From this case, we found that GALAC1 disease required multiple infusion treatments for a long time in the neonatal ward. This may be an important factor in the occurrence of *P. diazotrophicus* bloodstream infection for neonate. A diet that minimises galactose intake is the cornerstone of treatment for GALAC1 [7]. In the present study, a lactose-free milk powder diet improved the main symptoms of GALAC1 in the infant.

In recent years, reports of multidrug-resistant *P. diazotrophicus* have been increasing. Concerningly, the strains primarily carried carbapenem resistance genes, *bla*_*NDM−1*_ or *bla*_*KPC*,_ on plasmids resistant to most β-lactam antibiotics, which can easily cause horizontal transmission [1, 9]. Fortunately, no carbapenem resistance genes were found in this study, and meropenem treatment ultimately controlled the infection.

In conclusion, we successfully identified *P. diazotrophicus* in neonates with sepsis in China and isolated *P. diazotrophicus* from patients with galactosemia. Galactosemia may be an important cause of neonatal sepsis. These findings further expands the current understanding of the clinical characteristics of GALAC1.

## Data Availability

The datasets used during the current study available from the corresponding author on reasonable request.

## Abbreviations

P. diazotrophicus: Phytobacter diazotrophicus
WGS: Whole-genome sequencing
GALAC1: Galactosemia type 1
WES: Whole-exome sequencing
MLSA: Multilocus sequence analysis
dDDH: Silico DNA-DNA hybridisation
ANI: Average nucleotide identity
